# Comparison between manual and mechanical chest compressions during resuscitation in a pediatric animal model of asphyxial cardiac arrest

**DOI:** 10.1371/journal.pone.0188846

**Published:** 2017-11-30

**Authors:** Jorge López, Sarah N. Fernández, Rafael González, María J. Solana, Javier Urbano, Blanca Toledo, Jesús López-Herce

**Affiliations:** 1 Pediatric Intensive Care Department, Gregorio Marañón General University Hospital, Madrid, Spain; 2 Pediatrics Department, School of Medicine, Complutense University of Madrid, Spain; 3 Health Research Institute of the Gregorio Marañón Hospital, Madrid, Spain; 4 Red de Salud Maternoinfantil y del Desarrollo (Red SAMID) RETICS, Madrid, Spain; Waseda University, JAPAN

## Abstract

**Aims:**

Chest compressions (CC) during cardiopulmonary resuscitation are not sufficiently effective in many circumstances. Mechanical CC could be more effective than manual CC, but there are no studies comparing both techniques in children. The objective of this study was to compare the effectiveness of manual and mechanical chest compressions with Thumper device in a pediatric cardiac arrest animal model.

**Material and methods:**

An experimental model of asphyxial cardiac arrest (CA) in 50 piglets (mean weight 9.6 kg) was used. Animals were randomized to receive either manual CC or mechanical CC using a pediatric piston chest compressions device (Life-Stat®, Michigan Instruments). Mean arterial pressure (MAP), arterial blood gases and end-tidal CO2 (etCO2) values were measured at 3, 9, 18 and 24 minutes after the beginning of resuscitation.

**Results:**

There were no significant differences in MAP, DAP, arterial blood gases and etCO2 between chest compression techniques during CPR. Survival rate was higher in the manual CC (15 of 30 = 50%) than in the mechanical CC group (3 of 20 = 15%) p = 0.016. In the mechanical CC group there was a non significant higher incidence of haemorrhage through the endotracheal tube (45% vs 20%, p = 0.114).

**Conclusions:**

In a pediatric animal model of cardiac arrest, mechanical piston chest compressions produced lower survival rates than manual chest compressions, without any differences in hemodynamic and respiratory parameters.

## Introduction

Resuscitation guidelines are based on international consensus. Recent guidelines emphasize the importance of good-quality chest compressions during cardiopulmonary resuscitation (CPR) [1,2,3]. Depth, pressure, duty cycle, release, hands-off time and ventilation determine the quality of chest compressions.

Some clinical and laboratory studies in adults and children show that the quality of chest compressions is often suboptimal, even when delivered by healthcare professionals or after a CPR course [3,4,5]. One of the most important problems with manual chest compressions is that rescuers may not have the strength to perform sufficient compression depth and, consequently, mean arterial pressure (MAP), diastolic arterial pressure (DAP) and coronary perfusion pressure (COPP) are low. Moreover, quality of chest compressions deteriorates with time during CPR, probably due to rescuer fatigue [6,7].

Mechanical thoracic compression devices have certain advantages over manual CC: they provide a stable frequency, depth and duration of compression-decompression of the chest. Thus, the characteristics of chest compressions remain stable over a long period of time and, since the rescuer doesn’t have to deliver the chest compressions, he can focus on other important duties during CPR [8,9]. These machines use either an automatic piston or a band-like mechanism.

Several studies in adults and in adult animal models show that mechanical compression devices more effectively achieved process goals than manual compression, such as a higher MAP and DAP with fewer interruptions of chest compressions [10,11]. However, most of the clinical studies in adults have not demonstrated that mechanical chest compressions achieve greater return of spontaneous circulation or survival than manual chest compressions [12,13,14,15,16,17].

There is no experience in children with mechanical chest compressions because all the commercially available devices are approved for adults only. Before considering the possibility of conducting a clinical study, it is essential to assess the efficacy and potential harm of mechanical devices in pediatric animal models.

The hypothesis of our study was that piston point-compression mechanical CC achieves better MAP and DAP and higher return of spontaneous circulation (ROSC) than manual CC in a pediatric animal model of cardiac arrest.

## Material and methods

We conducted a randomized controlled experimental clinical trial in 50 Maryland piglets that were genetically identical. The study was approved by the Local Ethics Committee for Animal Research of Gregorio Marañón General Universitary Hospital and was carried out by qualified staff. International guidelines for ethical conduct in the care and use of experimental animals were applied throughout the study.

Animals were housed for 24 hours before the experiment and were fasted overnight (with free access to water). Piglets were pre-medicated with intramuscular ketamine (15 mg/kg) and atropine (0.02 mg/kg) before obtaining a peripheral venous access. After starting continuous cardio-respiratory monitoring, a single dose of iv propofol (5 mg/kg), fentanyl (5 mcg/kg) and atracurium (0.5 mg/kg) were administered for orotracheal intubation, followed by a continuous intravenous infusion of propofol (10 mg/kg/h), fentanyl (10 mcg/kg/h) and atracurium (2 mg/kg/h). Fluids with glucose and saline at 20 ml/hr was maintained throughout the experiment.

Piglets were mechanically ventilated (Servo 900C^®^ Ventilator, Siemens-Elema, Solna, Sweden) with the following initial settings: tidal volume 10 ml/kg, 20 bpm, PEEP 4 mmHg, FiO_2_ 0.45, to assure no hypoxia previously to cardiac arrest induction. Settings were adjusted to obtain an end-tidal CO_2_ (etCO_2_) between 30–40 mmHg and an arterial CO_2_ pressure between 35 and 45 mmHg.

Continuous monitoring of the following parameters were registered: electrocardiogram (ECG), transcutaneous oxygen saturation (HeartStart XL+^®^, Philips Medical Systems, Andover, Massachusetts, USA).

Cannulation of femoral arterial and venous accesses used ultrasound guidance. A three-lumen 5F catheter was used for continuous central venous pressure (CVP) monitoring, blood sample extraction and drug infusion. A 4F PiCCO^®^ catheter (PiCCO^®^, Pulsion Medical System, Munich, Germany) for monitoring arterial pressure was placed in the contralateral femoral artery. Blood gas analyses were processed in a GEM Premier 300^®^ gas analyzer (Instrumentation Laboratory, Lexington, Kentuky, USA).

After a 30-minute stabilization period, baseline data were collected and arterial and venous blood gases were drawn to assess ventilation and oxygenation.

Asphyxial cardiac arrest was induced by disconnecting the piglets from the ventilator for 10 minutes after receiving an additional bolus of atracurium (0.5 mg/kg) [18,19,20,21,22]. Time to cardiac arrest was registered. Cardiac arrest was defined as a mean arterial pressure (MAP) under 25 mmHg as has been described previously [18,19,20,21,22]. After 10 minutes of asphyxia all pigs were in cardiac arrest. At this moment data including monitoring parameters and blood gases were recorded and then resuscitation was started. At this point, animals were randomized into one of the two therapeutic groups: mechanical CC and manual CC with a relation of 1 to 1.5 respectively. Our hypothesis was that mechanical device produces better chest compression and higher return of spontaneous circulation (ROSC). So we designed a randomization with less number of animals in the mechanical chest compression group to try to reduce the number of animals used in the study. Then, advanced resuscitation was initiated: the animal was connected to the ventilator (with the same parameters as before the disconnection, except for a FiO_2_ of 1.0).

In both groups continuous chest compression was performed. Manual chest compressions were guided by a metronome-tailored rate of 100 compressions per minute (cpm). We tried to compress about 1/3 of the antero-posterior chest diameter, but depth of chest compression was not measured. A mechanical device, Thumper mechanical CPR, model 1007/CC specially adapted for pediatric size (Michigan Instruments, USA) was used. This is a piston mechanical chest compression device that allows adjustment of compression depth from 0 to 8 cm and compression rate. Duty cycle is preset for 50/50 cycle (50% compression and 50% release). Mechanical chest compression was programmed to a rate of 100 cpm. Compression depth was adjusted before cardiac arrest in each animal to achieve 1/3 of the anteroposterior diameter of the chest. The same depth was maintained throughout the experiment.

Pulse and ECG were assessed at 3 minute intervals, during less than 10 seconds, and the provider delivering chest compressions was swapped to avoid fatigue. Adrenaline (0.02 mg/kg/dose) was administered every 3 minutes and sodium bicarbonate (1 mEq/kg/dose) at 9 and 18 minutes of CPR. Animals were defibrillated (4 J/Kg) if a shockable rhythm was identified; adrenaline and amiodarone (5 mg/kg) were administered after the third defibrillation.

The following parameters were continuously monitored and collected at baseline and every 3 minutes after the initiation of CPR: Heart rate and rhythm, systolic arterial pressure (SAP), diastolic arterial pressure (DAP), mean arterial pressure (MAP), central venous pressure (CVP) and etCO_2._ Arterial blood gases were drawn at baseline, 10 minutes after asphyxia and at 3, 9, 18 and 24 minutes after the beginning of the resuscitation. S1 Table

Resuscitation was discontinued upon ROSC or after 24 minutes of CPR. Broncho-pulmonary hemorrhaghe was defined as red gross blood appearing in the tracheal tube. Animals achieving ROSC were later sacrificed by means of propofol and potassium chloride overdose. Necropsy was performed in several but not all cases, finding that hemorrhage through the tracheal tube corresponded to lung injury without macroscopic evidence of cardiac injury.

SPSS 21.0 (IBM SPSS Statistics, Chicago, Michigan, USA) was used for statistical analysis. Variables followed a normal distribution according to the Kolmogorov-Smirnov test. Continuous variables are expressed as mean and standard deviation and categorical variables as absolute percentages. Student-T test and chi-squared (χ^2^) were used to compare continuous and categorical variables. Statistical significance was defined as p<0.05.

## Results

50 piglets between 1 and 2 months of age weighing between 9 and 11 kg were studied. They were randomized into two groups: Group 1) mechanical (20 piglets); Group 2) manual (30 piglets). Baseline characteristics and after 10 minutes of asphyxia of the two groups are shown in Table 1. There were no statistically significant differences between groups.

**Table 1:** Comparison of parameters between manual and mechanical chest compression groups at baseline and at 10 minutes of asphyxia before the beginning of cardiopulmonary resuscitation.

Parameter	Manual chest compressions Mean SD	Mechanical chest compressions Mean SD	p
Weight (kg)	9.9 1.8	9.9 0.9	.871
Time to CA (minutes)	7.1 1.3	6.9 1.2	.661
Basal Heart rate (bpm)	110.0 26.8	111.3 28.6	.877
Asphyxia Heart rate (bpm)	63.4 18.9	58.4 22.5	.520
Basal MAP (mmHg)	78.7 13.4	71.9 14.8	.101
Asphyxia MAP (mmHg)	12.9 5.3	14.7 4.8	.336
Basal pH	7.47 0.03	7.45 0.03	.112
Asphyxia pH	7.12 0.08	7.09 0.07	.194
Basal PaO_2_ (mmHg)	155.5 46.8	134.3 41.6	.113
Asphyxia PaO_2_ (mmHg)	12.4 4.4	13.2 4.9	.546
Basal PaCO_2_ (mmHg)	41.7 4.7	44.0 3.5	.071
Asphyxia PaCO_2_ (mmHg)	84.3 15.1	85.7 13.3	.751
Basal arterial SatO_2_ (%)	98.8 1.8	98.7 1.1	.297
Asphyxia arterial SatO_2_ (%)	8.2 5.0	7.8 5.1	.838
Basal Lactate (mmol/L)	0.8 0.4	0.7 0.2	.161
Asphyxia Lactate (mmol/L)	5.7 1.4	5.7 1.6	.994

Asphyxia: 10 minutes of asphyxia. CA: cardiac arrest; MAP: mean arterial pressure. PaO_2_: oxygen arterial pressure; PaCO_2_: CO_2_ arterial pressure; SD: standard deviation.

Forty-seven piglets (94%) had non-shockable rhythms 10 minutes after asphyxia, with no significant differences between groups (p = 0.265).

**Fig 1:**
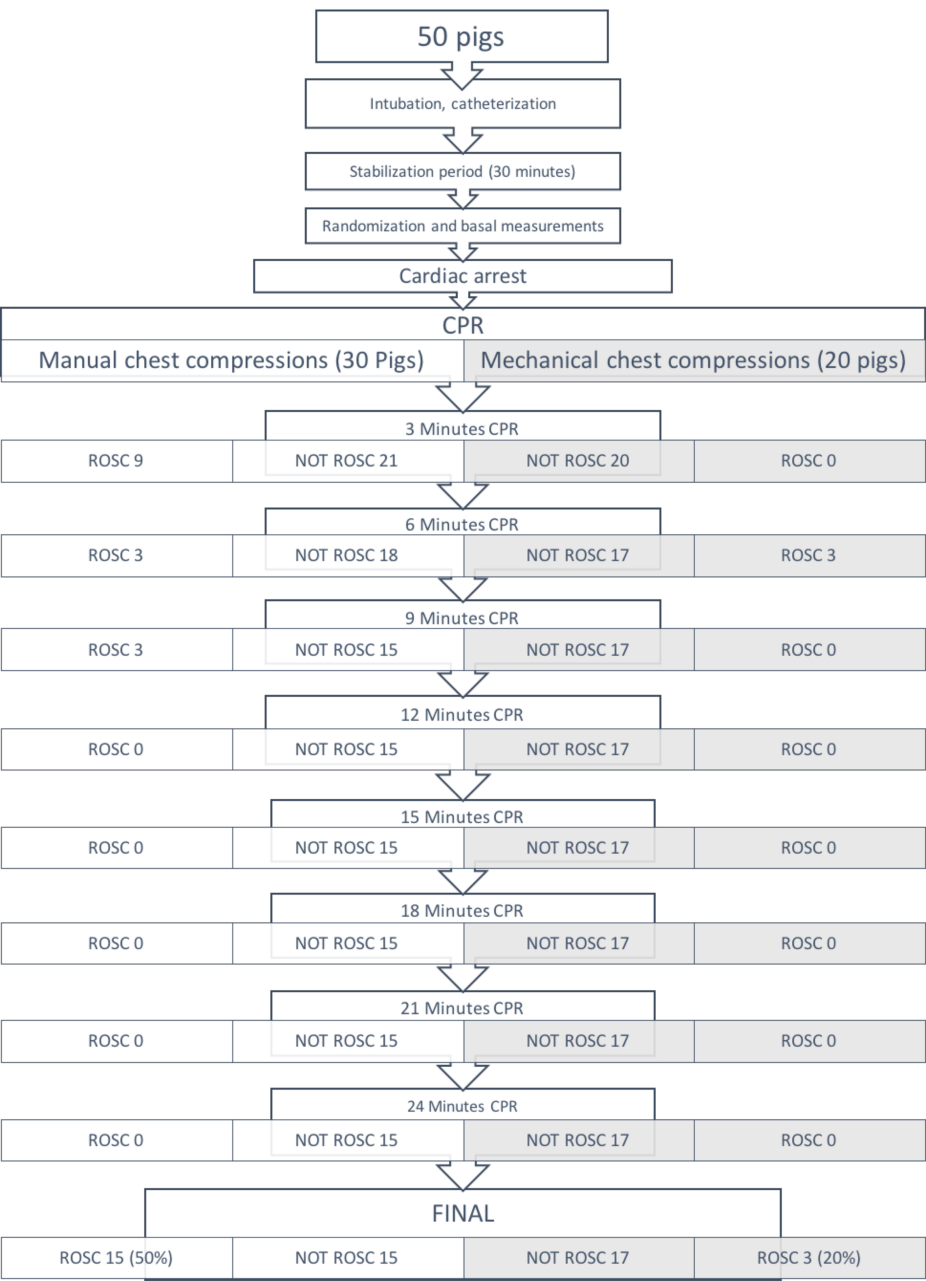
Evolution of animals in each group.

After cardiopulmonary resuscitation, 18 piglets achieved ROSC (36%). The percentage of ROSC was significantly higher in the manual chest compression group, 15 of 30 (50%), than in the mechanical chest compression 3 of 20 (15%), p = 0.012. Fig 1 showed the evolution of animals in each group. When only animals without hemorrhage were analyzed, ROSC was higher in manual CC group (15 of 24) 62.5% than in mechanical CC group (3 of 11) 27.7%, but there were not sufficient animals to reach statistical significance p = 0.053.

Only 3 pigs had ventricular fibrillation at the start of cardiac arrest and another 18 in some moment of CPR (without differences between groups 12 manual CC and 9 mechanical CC). Only 3 of these animals reached ROSC (2 in manual CC and one in mechanical chest compression).

**Fig 2:**
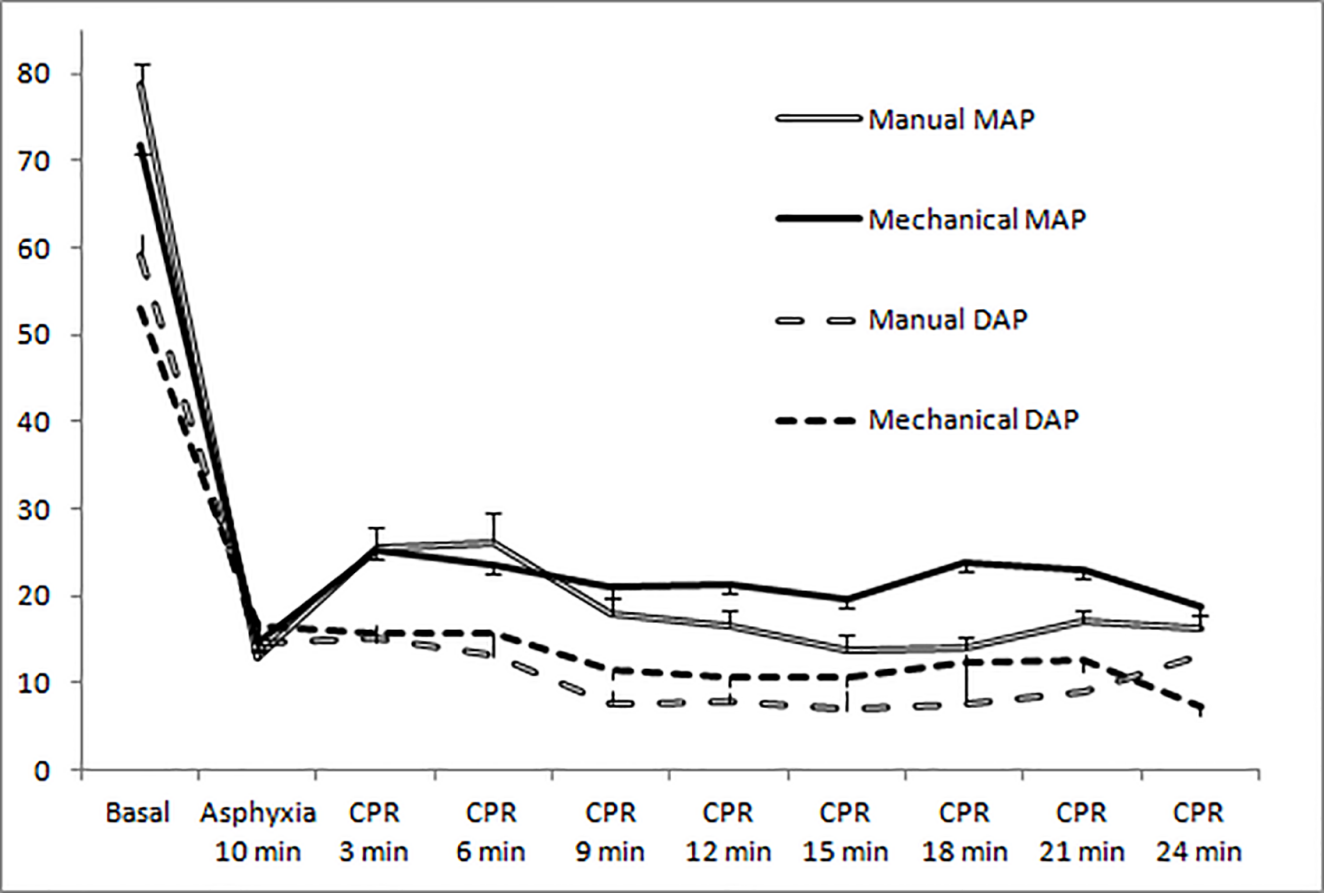
Evolution of arterial pressure in both resuscitation groups. (Mean and standard error of the mean). There were significant differences at 15 minutes (p = 0.036), and 18 minutes (p = 0.006) of cardiopulmonary resuscitation.

Fig 2 shows the evolution of arterial pressure in both groups. It trended higher in the mechanical chest compression group after 9 minutes of resuscitation, but was significantly higher only in MAP at 15 and 18 minutes. There were non-significant differences in diastolic arterial pressure.

Comparison of MAP, DAP and CVP between ROSC and non-ROSC piglets was performed. Only MAP and CVP at six minutes of CPR were statistically significantly higher in survival animals; MAP 42.7 (16.0) versus 22.6 (11.6) mmHg p = 0.004 and CVP 20.2 (8.7) versus 20.2 (8.7) mmHg, p = 0.032.

**Fig 3:**
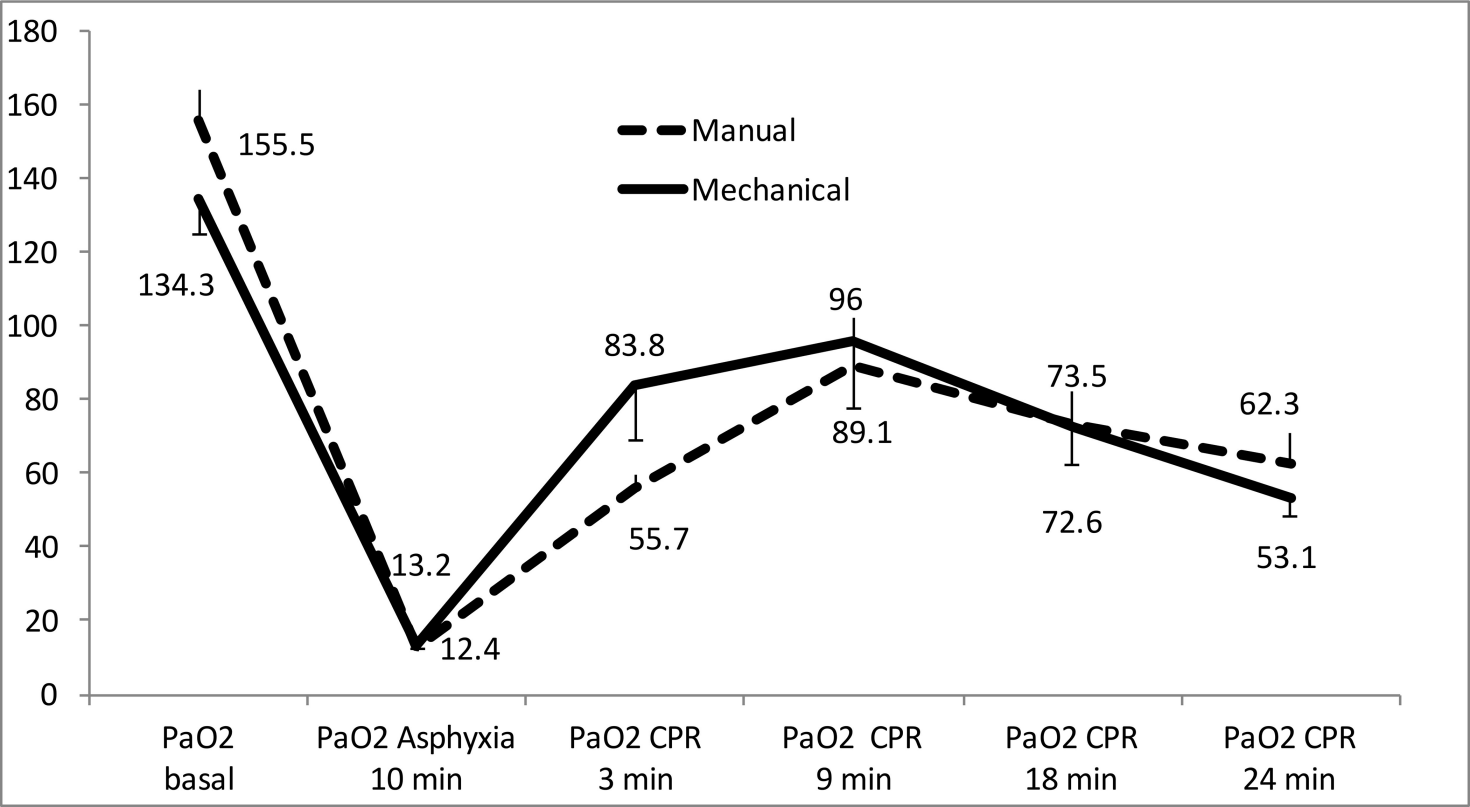
Evolution of arterial PO_2_ mean values (mmHg) in both resuscitation groups. There were no significant differences between groups. (Mean and standard error of the mean).

**Fig 4:**
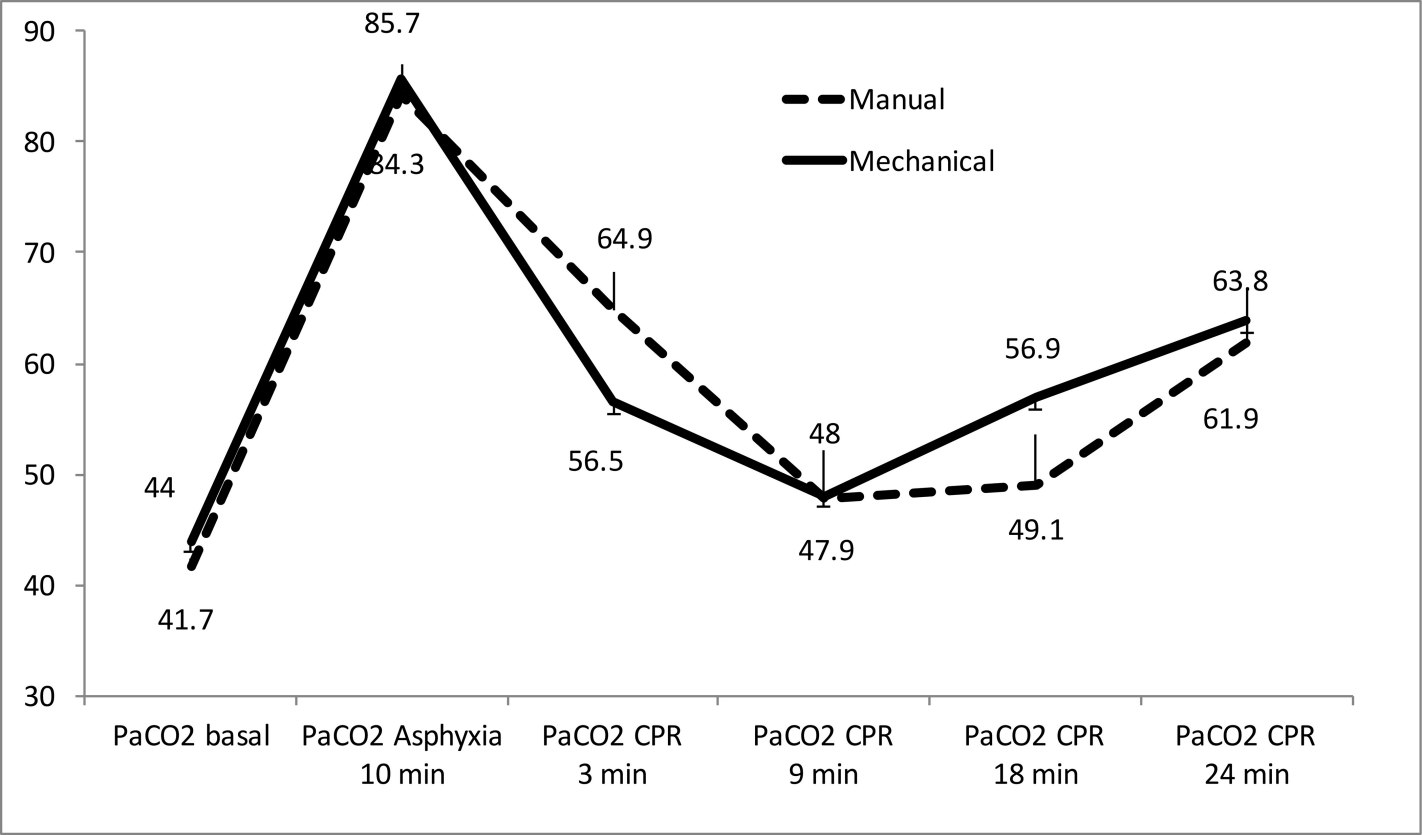
Evolution of arterial PCO_2_ mean values (mmHg) in both resuscitation groups. (Mean and standard error of the mean). There were only significant differences at 3 minutes of resuscitation p = 0.032.

**Fig 5:**
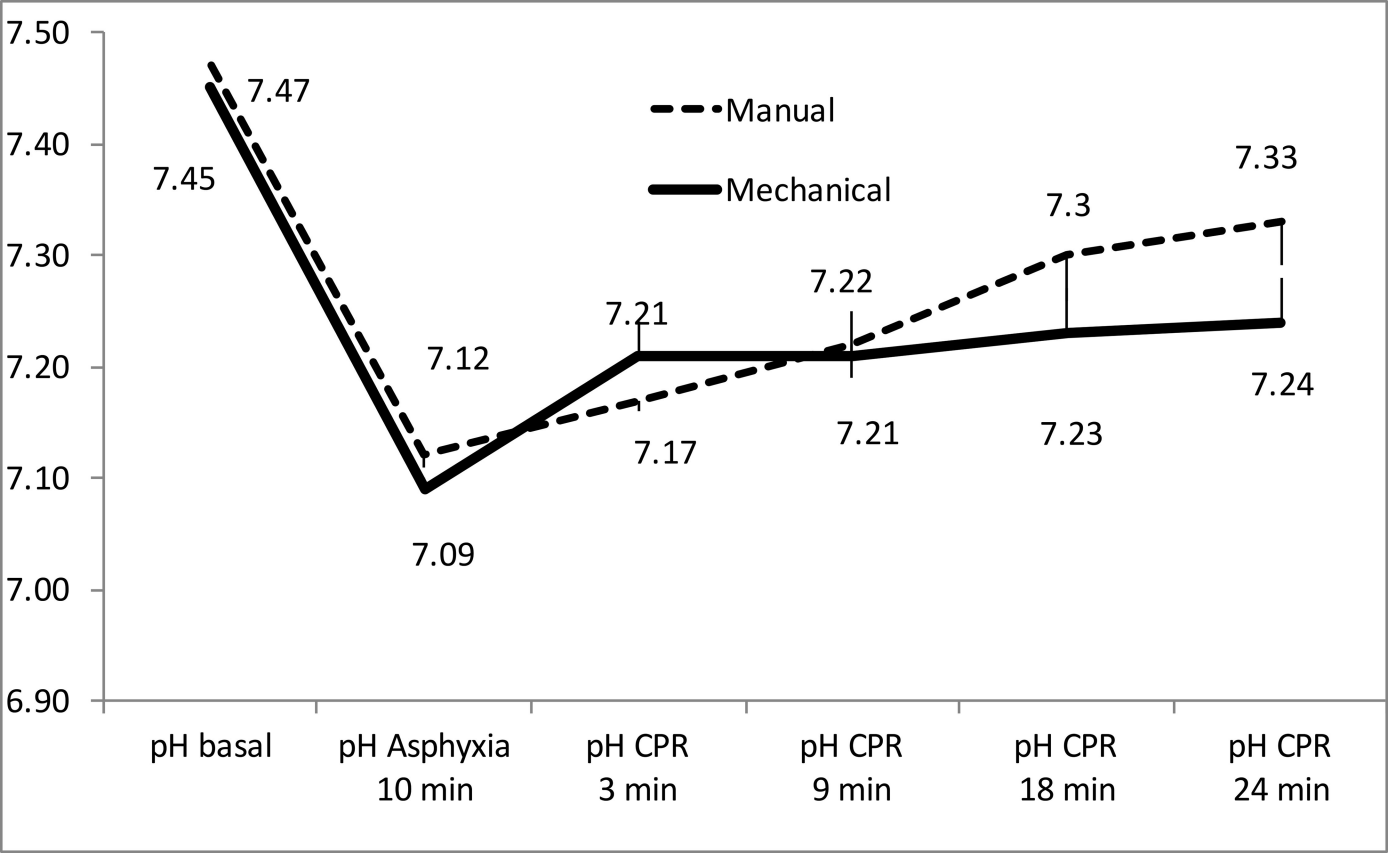
Evolution of pH mean values in both resuscitation groups. (Mean and standard error of the mean).

**Fig 6:**
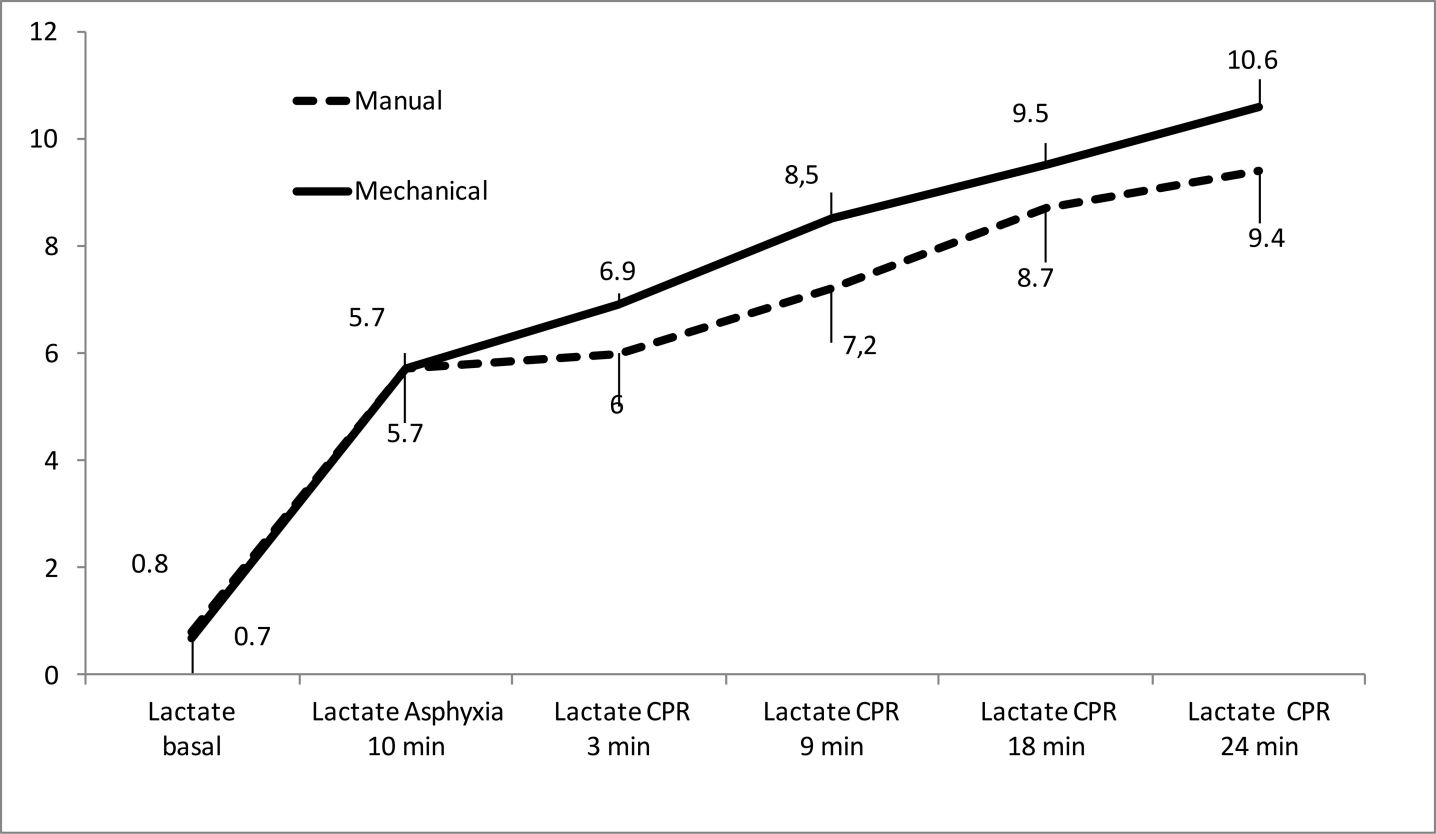
Evolution of lactate mean values (mmol/L) in both resuscitation groups. (Mean and standard error of the mean). There were only significant differences at 3 minutes of resuscitation (p = 0.002).

Figs 3 to 5 show the evolution of arterial blood gases and lactate concentration increased throughout experiment. PaO_2_ increased significantly over the first 9 minutes of CPR and then dropped modestly. There were only significant differences between groups at 3 minutes of resuscitation (Fig 3). PaCO_2_ decreased over the first 9 minutes of CPR, then increased slightly without statistically significant differences between both groups (Fig 4). pH increased over the course of CPR without significant differences between the two groups (Fig 5). Lactate increased throughout CPR. It was slight higher in the mechanical compression group, with significant differences at 3 minutes of resuscitation (Fig 6).

**Fig 7:**
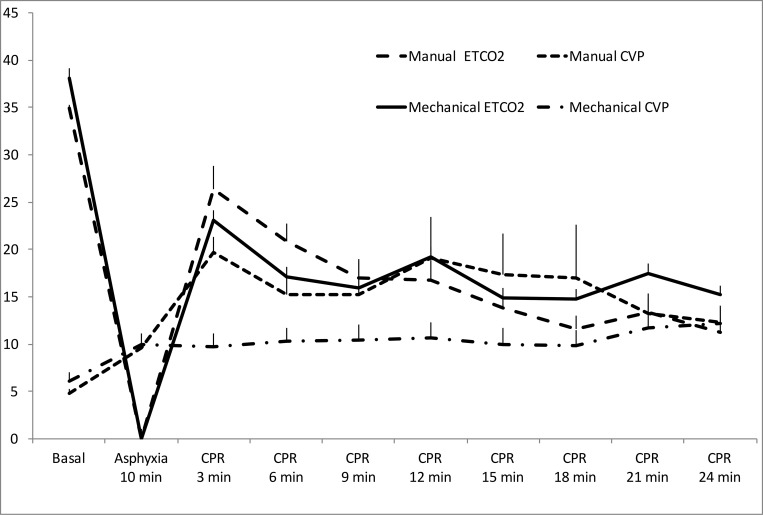
Evolution of central venous pressure and ETCO2 (Mean and standard error of the mean).

In mechanical chest compression group, mean CVP was lower, but without statistically significant differences (Fig 7). There were no significant differences in EtCO2 (Fig 7).

### Secondary effects

15 animals (30%) bled through the endotracheal tube. The incidence of hemorrhage was higher in the mechanical CC group (9/20 = 45%) than in the manual CC group (6/30 = 20%) but the differences were not statistically significant (p = 0.059). The haemorrhage showed up between 6 and 18 minutes of resuscitation.

51% of the animals without a hemorrhage survived but none of animals with a haemorrhage did (p = 0.001). Necropsy was performed in 4 of the animals with pulmonary bleeding all of which had hemorrhagic parenchymal lung injury.

## Discussion

Our study is, to our knowledge, the first to analyze the effect of mechanical and manual chest compression during CPR on oxygenation, ventilation, hemodynamics, tissue perfusion and ROSC in a pediatric animal model of asphyxial cardiac arrest.

The results from this study offer some valuable information:

Our results show that mechanical chest compressions in an infant animal model, although producing a slightly higher blood pressure, especially when CPR is prolonged, achieve a lower frequency of ROSC than manual chest compressions.

There are no studies with mechanical chest compression in children, because there are no specific mechanical chest compression devices for children. Most studies in manikins, adult animal models and adults are performed with circumferential load distributing band devices. Some studies in adult manikins found that mechanical chest compressions with circumferential load distributing band are deeper and are performed correctly more often than manual chest compressions [8,9].

However, other studies with adult manikins show that these devices can provide poor-quality chest compressions due to failure to recognize and correct a malposition of the device that may counteract a potential benefit of mechanical chest compressions [23]. In our study, an investigator was continuously monitoring the position of the mechanical compressor to avoid malposition and maintain the depth of compression.

So, current data suggests that circumferential load distributing band mechanical devices may be used to deliver CPR because they achieved better physiological parameters, higher ROSC and less secondary effects than manual compressions [10]. Nevertheless, our study shows that hemodynamic, ventilation, oxygenation and tissue perfusion parameters were very similar between mechanical and manual chest compressions. As it happens in humans, the configuration and compliance of the thorax in the infant pig is different from that in the adult pig. This could explain the difference in physiological findings and in side effects.

Some clinical studies have suggested that the quality of CPR is also better with mechanical devices because they provide deeper compressions, CPR remains stable and there are fewer interruptions [11]. Other studies showed higher return of spontaneous circulation and survival to hospital discharge with mechanical chest compressions but did not reach statistical significance [14].

However, large randomized trials, systematic reviews and meta-analysis did not find significant differences in ROSC, survival and neurological outcome between mechanical devices and manual chest compressions [12,13,14,15,16,17]. Neither were there any differences between each of the mechanical devices and manual compressions [16], although only a few studies used piston-type devices like the one we used in our study [16,24]. Moreover, some clinical studies in adults have found that chest compression devices are not always efficient and have secondary effects [25].

A systematic review and meta-analysis of studies performed in in-hospital cardiac arrest found an association between the use of mechanical chest compression devices and an improved short-term and hospital survival. There was also evidence of improvements in physiological outcomes [26]. So, according to the data available at this moment, mechanical devices could be indicated to deliver chest compressions when manual CPR is difficult, such as during ambulance transport or prolonged CPR [17].

### Side effects

In our study, the use of mechanical chest compression devices was associated with a higher, although non-statistically significant, incidence of pulmonary hemorrhage, which may contribute to the lower observed survival since no pig that experienced pulmonary hemorrhage achieved ROSC.

Our findings do not corroborate what was found in experimental studies with adult pigs [27]. Xantos et al treated 106 swine (53 with a mechanical device (Lucas^R^) and 53 with manual chest compression). After autopsy, sternal fractures (18 versus 2, P = 0.003); rib fractures (16 versus 4, P = 0.001); liver hematomas (9 versus 2, P = 0.026); and spleen hematomas (8 versus 0, P = 0.003), were more frequent in the manual chest compression group. No lung or broncho-tracheal lesions were described [27].

No serious adverse effects were found with mechanical or manual chest compressions in most clinical studies in adults [15]. Some adult studies compared the rate of rib or sternal fractures and internal organ injury between mechanical devices and manual chest compression. Halperin [28] and Lu [24] found a lower incidence of rib or sternal fractures with pneumatic vest and Thumper mechanical devices but Taylor [29] found a higher incidence. Lu [24] and Taylor [29] found lower incidence of internal organ injury with mechanical devices.

In a recent retrospective cohort study in adults, post-mortem computed tomography was performed in patients after CPR [30]. Posterior rib fracture, hemoperitoneum, and retroperitoneal hemorrhage were more frequent in patients with load-distributing band Autopulse^R^ mechanical CPR. 6% had hemothorax but pulmonary haemorrhage was not described [30].

Another prospective forensic autopsy cohort study evaluated the prevalence and risk factors of intra-thoracic injuries associated with manual CPR in 80 patients. CPR-associated injuries were found in 93.7% of cases; the majority of injuries were skeletal chest fractures (rib fractures in 73.7%, sternal fractures in 66.3%). Intra-thoracic injuries were identified in 41.2% of cases. Contusion of at least one lung lobe was found in 31.2%, lung laceration in 2.5%, and hemothorax in 5.0% of cases. Transmural heart contusion was identified in 17.5% of cases; hemopericardium resulting from right atrium rupture or aortic rupture was revealed in 8.7% of cases [31].

In a prospective multicentre trial including 222 patients (83 manual CPR/139 Lucas^R^ mechanical CPR), autopsies were conducted after unsuccessful CPR. 75.9% of the patients in the manual CPR group and 91.4% of the ones in the mechanical CPR group (p = 0.002) suffered CPR-related injuries. Sternal fractures were present in 54.2% of the patients in the manual CPR group and in 58.3% in the mechanical CPR group (p = 0.56). The incidence of rib fractures was 64.6% of the manual CPR group and in 78.8% of the mechanical CPR group (p = 0.02) [32]. In our study, we did not compare the incidence of sternal and rib fractures between both types of chest compression. The differences in secondary effects might be due, in part, to the mechanism that the different mechanical devices use to apply chest compressions. Thumper device produces a point compression to the sternum like manual compression and other devices produce more circumferential thorax compression.

The keel configuration of the infant pig's thorax might have played a role in the production of greater sternal and costal skeletal injury, which would lead to an increased risk of intrathoracic injury [31]. However we cannot prove this hypothesis because no specific necropsy was performed.

On the other hand, the side effects produced by thoracic compression may be more important in children and animals in the pediatric age, because, due to the compliance of the rib cage, the pressure is more easily transmitted to the internal organs with greater probability of secondary injury.

A recent study reviewed 467 chest computed tomography scans (93 infants, 110 children, and 264 adults) and suggested that current pediatric guidelines for compression depth are too deep [33]. An excessively deep chest compression may also contribute to a higher incidence of broncho-pulmonary lesions without improving resuscitation results.

## Limitations

Our study has several limitations. First, even though our asphyxial pediatric cardiac arrest model has been validated and is very similar to what happens in human pediatric patients, results from experimental animal studies must be interpreted with caution.

We tried to maintain the characteristics of chest compression as similar in both groups. However, the depth of chest compression and duty cycle was only programmed in the mechanical device, and they were not measured in none of both groups. The chest compression device that we used, although it regulates the exerted compression, can cause a different deformation in the thorax of the pig than a child because the chest wall configuration and compliance are different. A Pig has a keel-shaped thorax and infants have a more rounded thorax.

This can make both hemodynamic effects and the risk of skeletal and lung injury different. The incidence of bleeding through the tracheal tube, although not statistically significant, was much higher in the mechanical chest compression group. Probably the number of animals included in the study was not sufficient to reach statistical significance. In the four autopsied piglets, this bleeding coincided with a macroscopic lung injury. However, we cannot know if all animals with hemorrhage through the tube had lung damage because a systematic autopsy study was not performed on all animals. For this reason we cannot clearly establish the causes of bleeding, nor compare the incidence of injuries in other organs between the two types of chest compressions.

## Conclusions

In a pediatric animal model of cardiac arrest, mechanical chest compressions with a Thumper device produced lower survival rates than manual chest compression, without any differences in hemodynamic and respiratory parameters.

## Supporting information

S1 Table Data base. (SAV)
